# Entropy Mapping Approach for Functional Reentry Detection in Atrial Fibrillation: An In-Silico Study

**DOI:** 10.3390/e21020194

**Published:** 2019-02-18

**Authors:** Juan P. Ugarte, Catalina Tobón, Andrés Orozco-Duque

**Affiliations:** 1 Grupo de Investigación en Modelamiento y Simulación Computacional (GIMSC), Universidad de San Buenaventura, 050010 Medellín, Colombia; 2 Materiales Nanoestructurados y Biomodelación (MATBIOM), Universidad de Medellín, 050026 Medellín, Colombia; 3 Grupo de Investigación e Innovación Biomédica (GI^2^B), Instituto Tecnológico Metropolitano, 050034 Medellín, Colombia

**Keywords:** atrial fibrillation, functional reentry, entropy maps, fractionated electrograms, Shannon entropy, approximate entropy, sample entropy

## Abstract

Catheter ablation of critical electrical propagation sites is a promising tool for reducing the recurrence of atrial fibrillation (AF). The spatial identification of the arrhythmogenic mechanisms sustaining AF requires the evaluation of electrograms (EGMs) recorded over the atrial surface. This work aims to characterize functional reentries using measures of entropy to track and detect a reentry core. To this end, different AF episodes are simulated using a 2D model of atrial tissue. Modified Courtemanche human action potential and Fenton–Karma models are implemented. Action potential propagation is modeled by a fractional diffusion equation, and virtual unipolar EGM are calculated. Episodes with stable and meandering rotors, figure-of-eight reentry, and disorganized propagation with multiple reentries are generated. Shannon entropy (ShEn), approximate entropy (ApEn), and sample entropy (SampEn) are computed from the virtual EGM, and entropy maps are built. Phase singularity maps are implemented as references. The results show that ApEn and SampEn maps are able to detect and track the reentry core of rotors and figure-of-eight reentry, while the ShEn results are not satisfactory. Moreover, ApEn and SampEn consistently highlight a reentry core by high entropy values for all of the studied cases, while the ability of ShEn to characterize the reentry core depends on the propagation dynamics. Such features make the ApEn and SampEn maps attractive tools for the study of AF reentries that persist for a period of time that is similar to the length of the observation window, and reentries could be interpreted as AF-sustaining mechanisms. Further research is needed to determine and fully understand the relation of these entropy measures with fibrillation mechanisms other than reentries.

## 1. Introduction

Among all of the cardiac arrhythmias, atrial fibrillation (AF) is the most recurrent in clinical practice [1]. It is estimated that AF has a worldwide prevalence of 3%, which is anticipated to continuously increase over the next several decades [2]. Patients with AF experience a decreased quality of life, and up to 40% of this population is hospitalized each year [1]. The costs resulting from treatment and complications reach 1% of the healthcare institutions budgets [3]. Thus, AF imposes a substantial socioeconomic burden on the world healthcare systems, and the problem is worsened in developing regions, such as Latin America [1,3,4]. AF is a disorder of the cardiac rhythm, characterized by a rapid and irregular electrical activation of the atria [5]. Its treatment includes pharmacological and surgical strategies. The former consists of rhythm control by means of drug administration, and the latter involves studying the electrophysiological properties of the atria by accessing the cardiac chambers with catheters. The goal of these invasive techniques is to revert the AF to sinus rhythm by ablating key zones over the atria [5]. Catheter ablation improves the success rate of sinus rhythm restoration compared with pharmacological therapy. In early-stage AF patients (paroxysmal AF), the ablation approach has a reported success rate of up to 70%. However, in more advanced stages of the arrhythmia (chronic AF), the overall success rates remain suboptimal [1].

There is strong evidence supporting the notion that focal ectopic activity at the pulmonary veins is a critical mechanism for sustaining some cases of AF [6,7]. Thus, the pulmonary veins are a widely accepted ablation target, although the efficiency is limited to paroxysmal scenarios [5]. Therefore, the aim of an electrophysiological study is to identify ablation targets that are related to the perpetuation of the AF. The catheter ablation procedure requires an electroanatomical mapping of the atrial chambers with the objective of assessing the electrical propagation and looking for arrhythmogenic mechanisms. This task involves recording electrograms (EGMs), which are electrical signals from the cardiac surface. EGMs and their spatial localization provide information about the underlying electrical activity, and the acquired data can serve to localize AF substrates. Therefore, the identification of arrhythmogenic mechanisms requires signal processing tools for EGM interpretation.

Frequency analysis constitutes the conventional approach for EGM analysis [8,9,10]. It looks for rapid activation rates that are related to ectopic foci [11]. The EGM morphology representing a focal source has a high degree of regularity. However, during an AF episode that is maintained by functional reentries, such as rotors (spiral wave turning around a pivot point), the chaotic propagation is reflected by an irregular EGM, and the frequency analysis poorly correlates with these activations [12]. In this regard, more than a decade ago, complex fractionated atrial electrograms (CFAEs) were postulated to be potential targets for ablation [13]. They were originally described according to temporal criteria. Although the initial reports indicated high success rates of procedures involving CFAE ablation, not all the subsequent studies were able to reproduce such outcomes, generating a controversy around the efficacy of CFAE ablation [14,15,16,17,18,19,20,21,22,23]. The broad and nonspecific CFAE definition and the dependence on the operator’s judgment are among the principal reasons for this failure [15,24]. To solve these limitations, novel computational tools for EGM analysis have been proposed, and the nonlinear dynamics theory plays an important role in describing EGM fractionation phenomena. Using nonlinear measures, new criteria for discriminating between fractionated and non-fractionated EGMs have been developed [25,26,27], and distinct degrees of fractionation can be quantified [28,29,30,31]. Moreover, studies looking for a relation between fractionated EGMs and arrhythmogenic mechanisms have been conducted [15,31,32,33,34,35,36,37,38,39]. The determination of such a relationship is relevant to the ablation procedure, and it could generate new insights into AF dynamics.

Bearing these ideas in mind, in this study, the entropy mapping approach is investigated for its use in detecting functional reentries. Functional reentries are hypothesized to play a leading role in sustaining AF [40]. Although this hypothesis remains controversial [41,42,43,44,45], there is growing evidence in its favor arising from quantitative causality analyses during AF [46,47,48] and rotor detection and ablation in humans [49,50,51]. Therefore, this work aims to characterize the reentry dynamics using three entropy measures: Shannon entropy (ShEn), approximate entropy (ApEn), and sample entropy (SampEn). The ShEn and ApEn have been previously applied to characterize stable functional reentries by means of EGM morphological irregularity [31,52], while SampEn has been applied to quantify and characterize EGM fractionation [30]. By implementing mathematical models, fibrillation episodes are simulated in 2D domains, and virtual EGMs are calculated, resembling electroanatomical mapping procedures. The morphological irregularity of fractionated EGMs are quantified by entropy values, and electroanatomical entropy maps are built. The obtained results show that the entropy maps are able to track reentry mechanisms under three distinct propagation dynamics, and ApEn and SampEn maps have appealing features for potential translation into a clinical context.

## 2. Materials and Methods

In this section, the methodological procedure for generating entropy maps from simulated atrial fibrillation episodes is presented. The mathematical model and formulas for simulating atrial fibrillation episodes and EGM signals are described. The entropy analysis of an EGM is outlined, including details of entropy estimation and the method for generating entropy maps.

### Model of Atrial Fibrillation

The ionic kinetics of the cardiomyocyte membrane are implemented using two mathematical models: the Courtemanche human atrial action potential model [53], which is a detailed biophysical model, and the Fenton–Karma model [54], which is a simplified model. For the reproduction of AF electrical remodeling in the Courtemanche formalism, the cholinergic effect is included by implementing the acetylcholine-dependent potassium current ($I_{KACh}$) as follows [55]:

(1) $$I_{KACh}=\left(\frac{10}{1+\frac{9.13652}{{\left[ACh\right]}^{0.477811}}}\right)\left(0.0517+\frac{0.4516}{1+e^{\frac{V+59.53}{17.18}}}\right),$$

where *V* is the membrane potential and [ACh] is the acetylcholine concentration. Additionally, the Courtemanche model is modified in order to simulate the electrophysiological conditions of paroxysmal (pAF) and chronic AF (cAF). To reproduce the electrical remodeling generated by the paroxysmal AF condition [56,57], changes in the ionic conductance of different ionic channels are incorporated: the maximum conductance of the transient potassium current, the ultrarapid outward potassium current, and the L-type calcium current are reduced by 25%, 25%, and 35%, respectively. To reproduce the electrical remodeling of isolated myocytes from patients with cAF [58,59,60,61], the ionic conductance of different ionic channels is adjusted: the maximum conductance of the ultrarapid outward potassium current and the L-type calcium current are reduced by 35%, the maximum conductance of the ultrarapid outward potassium current is reduced by 50%, and the maximum conductance of the inward rectifier potassium current is increased by 100%. Additionally, to simulate the early and late stages of cAF, 5 nM and 500 nM of acetylcholine are applied, respectively. The Fenton–Karma model is parameterized according to Set 1 from [54].

## 3. Model of Action Potential Propagation

In a previous work [62], the action potential propagation over a structurally heterogeneous 2D domain is proposed to be modeled by the following fractional diffusion equation:

(2) $$K\left(\frac{\partial^{\alpha_{x}}}{\partial x^{\alpha_{x}}}+\frac{\partial^{\alpha_{y}}}{\partial y^{\alpha_{y}}}\right)V\left(x,y,t\right)=C_{m}\frac{\partial V(x,y,t)}{\partial t}+J_{ion},$$

where *V* is the transmembrane potential, $J_{ion}$ represents the membrane currents described by the ionic kinetics, *K* is the diffusion coefficient, $C_{m}$ is the cellular membrane capacitance, and $\alpha_{x},\alpha_{y}\in \left[1.1,2\right]$ are the orders of horizontal and vertical partial fractional derivatives, respectively. For $\alpha_{x}=\alpha_{y}=2$, the standard diffusion equation is recovered. The value of *K* is defined for obtaining a conduction velocity of 67 cm/s [63] for the standard diffusion case.

A two-dimensional (2D) model of human atrial tissue is designed as a 4×4 cm^2^ surface, which is discretized into a 128×128 mesh. The spatial resolution is 312.5 μm. The cellular electrophysiological models are integrated into the 2D virtual tissue. Discretization and the numerical solution of Equation (2) is accomplished using a semi-spectral approach previously reported [64], using a time step of 0.01 ms.

The following conditions are simulated:

- pAF: Courtemanche pAF conditions without acetylcholine, $\alpha_{x}=\alpha_{y}=2$.
- cAF1: Courtemanche cAF conditions with 5 nM acetylcholine, $\alpha_{x}=\alpha_{y}=2$.
- cAF2: Courtemanche cAF conditions with 500 nM acetylcholine, $\alpha_{x}=\alpha_{y}=2$.
- cAF3: Courtemanche cAF conditions with 500 nM acetylcholine, $\alpha_{x}=2$ and $\alpha_{y}=0.85\alpha_{x}$.
- cAF4: Fenton–Karma cAF conditions, $\alpha_{x}=1.5$ and $\alpha_{y}=0.85\alpha_{x}$.
- cAF5: Fenton–Karma cAF conditions, $\alpha_{x}=1.4$ and $\alpha_{y}=0.85\alpha_{x}$.

### 3.1. Stimulation Protocols

**Figure 1:**
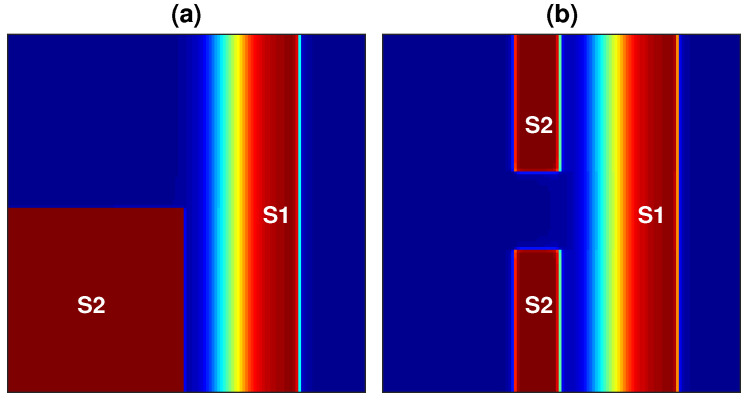
(**a**) Stimulation protocol (i): the S2 stimulus occurs after S1, and it is applied to the inferior left corner of the domain. (**b**) Stimulation protocol (ii): S2 occurs after S1 and consists of two stimuli applied to the middle portion of the domain. For both protocols, S1 is a plane stimulus applied to the left boundary, and it generates a plane wave traveling from left to right.

Reentry propagation patterns are generated by applying the S1–S2 cross-field stimulation protocol. S1 is a plane stimulus applied to the left border of the tissue by stimulating a region of 128 × 1 nodes, and this induces a plane wave that propagates over the entire tissue from the left side of the domain to its right boundary. Once this wave has passed over the first half of the domain, a second stimulus (S2) is applied in two configurations: (i) the S2 stimulus is applied to the first quarter of the domain by stimulating a region of 64 × 64 nodes, as shown in Figure 1a; (ii) the S2 stimulus consists of two stimuli applied to the middle portion of the tissue by stimulating two regions of 50 × 1 nodes, as shown in Figure 1b. Protocol (i) generates a single rotor, and protocol (ii) generates a figure-of-eight reentry pattern. Each stimulus consists of a rectangular pulse with a duration of 2 ms and a current of 4200 pA.

### 3.2. Virtual Electrograms

Unipolar EGMs at the atrial surface are simulated as the extracellular potential ($\Phi_{e}$) given by the following equation:

(3) $$\Phi_{e}=-\frac{1}{4\pi }\frac{\sigma_{i}}{\sigma_{e}}\;∭\;\nabla V\left(r\right)\cdot \nabla \left(\frac{1}{r}\right)dv,$$

where $\sigma_{i}$ and $\sigma_{e}$ are the intracellular and the extracellular conductivity, respectively; *r* is the distance from the source point to the virtual electrode position; and dv is the volume differential. The virtual electrodes are located 1 mm above the domain, and the EGMs are computed every millisecond for all nodes of the atrial domain.

### 3.3. Entropy Maps

In order to quantify the morphological irregularity of the EGM signals, an entropy-based approach is applied. The ShEn, ApEn, and SampEn measures are estimated by following the considerations incorporated in previous studies involving intracardiac EGMs [31,52]. Given an *N*-point EGM signal $x_{i}$, it can be described by a probability distribution *P*. If the distribution *P* is defined according to a voltage histogram built from $x_{i}$ with a fixed bin amplitude *b*, ShEn is calculated as follows:

(4) $$ShEn\left(N,b\right)=-\sum_{k=0}^{M-1}p_{k}\log_{2}p_{k},$$

where *M* is the number of histogram amplitude bins, and $p_{k}$ is the probability that a signal sample belongs to the *k*th amplitude bin.

Let the signal $x_{i}$ be considered as an *N*-dimensional point, and the parameter m:m<N is defined in such a way that *m*-dimensional points $x_{i,m}$ can be established from consecutive points of $x_{i}$. Let $B_{i}$ be the number of points $x_{j,m}$ that are arbitrarily close to $x_{i,m}$, and let $A_{i}$ be the number of points $x_{j,m+1}$ that are arbitrarily close to $x_{i,m+1}$. The arbitrarily close criteria are defined using the parameter *r*. Thus, ApEn is determined as follows [65]:

(5) $$ApEn\left(N,m,r\right)=-\frac{1}{N-m}\sum_{k=1}^{N-m}\log\left(\frac{A_{k}}{B_{k}}\right),$$

where the ratio $A_{i}/B_{i}$ estimates the conditional probability that a point $x_{j,m+1}$ is close to $x_{i,m+1}$ given that $x_{j,m}$ is close to $x_{i,m}$. Cases where i=j (self-matches) are included in order to avoid the indeterminate form.

On the other hand, SampEn is determined as follows [66]:

(6) $$SampEn\left(N,m,r\right)=-\log\left(\frac{\sum_{k=1}^{N-m}A_{k}}{\sum_{k=1}^{N-m}B_{k}}\right)=-\log\left(\frac{A}{B}\right),$$

where the ratio A/B estimates the conditional probability that an (m+1)-dimensional point is close, according *r*, to another (m+1)-point, given that this occurs for the *m*-dimensional case.

For numerical estimations of the presented entropy measures, their parameters must be defined. According to previous studies, for ShEn, b=0.01 [52]; for ApEn and SampEn, m=3 and r=0.38SD, where SD is the standard deviation of signal $x_{i}$ [31]. All measures are calculated for N=1000 according to [31]. The entropy measures are calculated for all EGMs recorded within a fibrillation episode simulation. Each signal of 1000 samples in length is represented by a single entropy value. Thus, an entropy map over the atrial domain can be generated.

### 3.4. Phase Map and Phase Singularity

Functional reentry dynamics can be well characterized by tracking the tip of the reentry. A functional reentry circulates around an excitable but unexcited core. The tip is the point around which the reentry circulates during a single spin. After several spins, the tip can change its position, defining a trajectory that is referred to as the reentry core. The instantaneous phase is calculated from the simulated unipolar atrial EGM in order to obtain the phase singularity (PS) that corresponds to the rotor tip. For this purpose, the Hilbert transform is applied, and a reconstructed sinusoidal signal with a period associated with the EGM cycle length is defined [67]. The PS is the point around which the phase changes by 2π (a complete cycle between −π and π) [68]. The PS trajectory is used as a reference to assess the ability of the entropy maps to characterize the reentry dynamics.

## 4. Results

The AF episodes are simulated by combining the physiological conditions and the stimulation protocols described in the previous section. All simulations had a duration of 4000 ms. Three distinct fibrillatory scenarios are studied: single rotor, figure-of-eight reentry, and multiple reentries. Entropy maps are built for each fibrillation episode and compared with the PS trajectory.

### 4.1. Single Rotor

**Figure 2:**
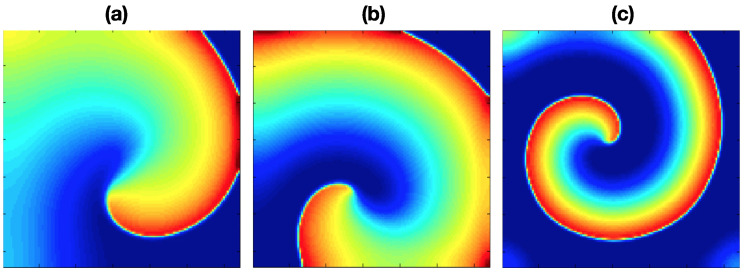
Rotor propagation patterns generated by applying stimulation protocol (i) and (**a**) the paroxysmal atrial fibrillation (pAF) condition, (**b**) chronic AF 1 (cAF1) condition, (**c**) cAF2 condition. The maps correspond to the last 1000 ms of each simulation.

Three fibrillation episodes were generated, and the rotor stability presents different behavior in each episode. These propagation patterns are achieved by applying stimulation protocol (i) under pAF, cAF1, and cAF2 conditions. Each simulation lasted 4000 ms. Figure 2 shows the propagation patterns generated for the three AF conditions.

**Figure 3:**
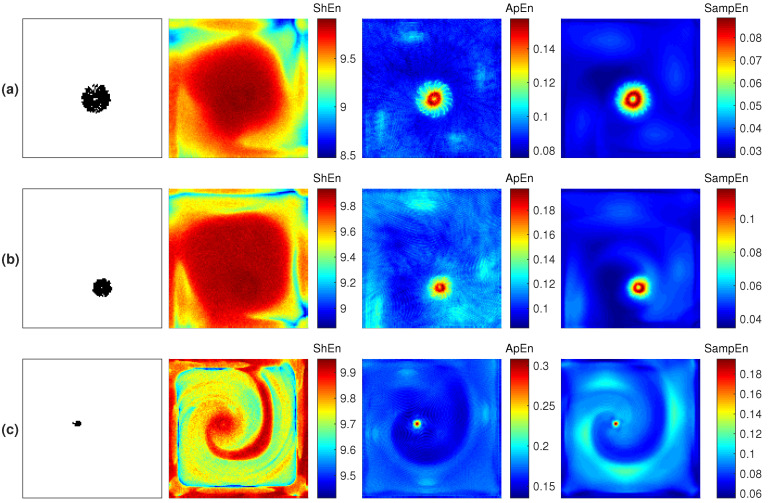
Reentry dynamics characterization maps corresponding to the rotors generated by applying stimulation protocol (i) and (**a**) pAF condition, (**b**) cAF1 condition, (**c**) cAF2 condition. The phase singularity (PS) trajectory is shown in the first column; Shannon entropy (ShEn), approximate entropy (ApEn), and sample entropy (SampEn) maps are shown from the second to the last column, respectively.

Figure 3a,b present the rotor tip spatial characterization for the three fibrillation conditions. The results corresponding to the last 1000 ms are presented. The first column shows the PS trajectory. On the basis of the core size depicted by the PS trajectory, three types of rotors are defined: meandering, mid-meandering, and stable. The pAF condition generates the larger core, named meandering (Figure 3a). The cAF1 condition generates a smaller core, named mid-meandering (Figure 3b). The cAF2 condition generates the smallest core, which depicts a stable reentry (Figure 3c). The third, fourth, and fifth columns depict the ShEn, ApEn and SampEn maps, respectively. It can be seen that the ShEn map is least able to describe the rotor tip. The ApEn and SampEn maps present similar outcomes for highlighting the rotor tip through the highest entropy values. However, both measures present different mapping features at the periphery of the rotor core.

### 4.2. Figure-of-Eight Reentry

**Figure 4:**
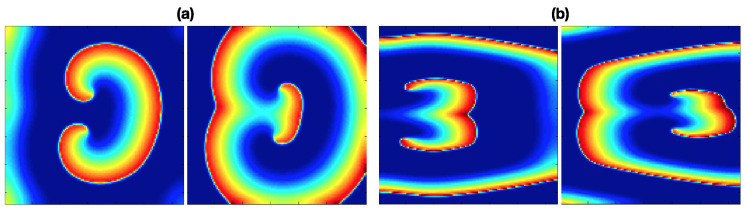
Figure-of-eight propagation patterns generated by applying stimulation protocol (ii) and (**a**) cAF2 condition, (**b**) cAF3 condition.

Two fibrillation episodes are generated by applying stimulation protocol (ii) with cAF2 and cAF3 conditions. Figure 4a,b depict the achieved propagation patterns for cAF2 and cAF3, respectively. Both simulations present a figure-of-eight reentry (two rotors rotating in opposite directions) with different dynamics. For cAF2, the rotors rotate around two stable pivot points, while for CAF3, the pivot points are migratory, depicting pivot lines.

**Figure 5:**
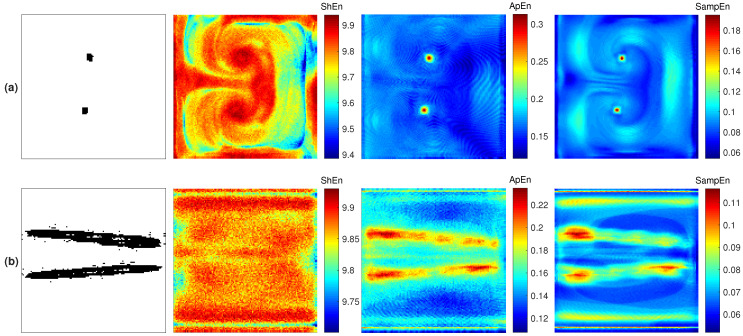
Reentry dynamics characterization maps corresponding to the figure-of-eight reentries generated by applying stimulation protocol (ii) and (**a**) cAF2 condition, (**b**) cAF3 condition. The PS trajectory is shown in the first column; ShEn, ApEn and SampEn maps are shown from the second to the last column, respectively.

Figure 5a,b present the maps characterizing the reentry dynamics. The PS trajectories (first column) indicate that the cAF2 condition generates a stable figure-of-eight reentry, and the cAF3 condition generates a figure-of-eight reentry that meanders through the middle part of the atrial domain. The ApEn and SampEn maps (second and third column) detect the reentry trajectory by presenting the highest entropy values, while the ShEn maps (first column) are not able to specifically detect the rotor tip. In cAF3 conditions, Figure 5b shows that the ApEn map better matches the linear trajectory of the reentry cores compared with the SampEn map. SampEn highlights two additional trajectories by middle entropy values at the top and bottom of the 2D domain. These can be interpreted as false detections. The ApEn map presents low and approximately uniform values at the periphery of the reentry.

### 4.3. Multiple Reentries

**Figure 6:**
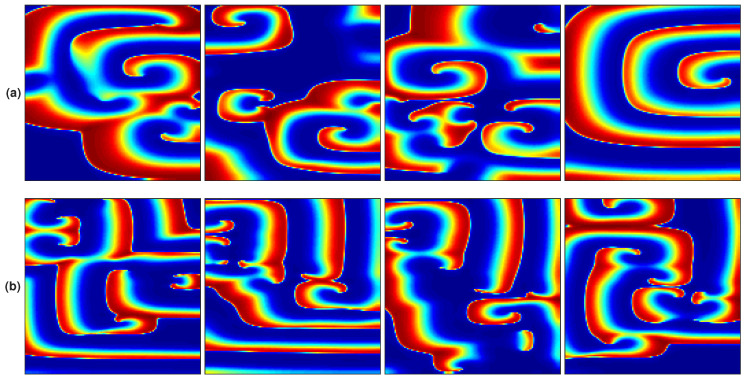
Multiple-reentry propagation patterns generated by applying stimulation protocol (i) and (**a**) cAF4 condition, (**b**) cAF5 condition. Four consecutive frames are presented in each case.

Two fibrillation episodes are generated by applying the stimulation protocol (i) with cAF4 and cAF5 conditions. Figure 6a,b present four consecutive frames from the resultant propagation patterns. At the beginning of the episode, in both cases, a single rotor is generated that breaks as the simulation progresses, leading to disorganized propagation with multiple small reentries.

**Figure 7:**
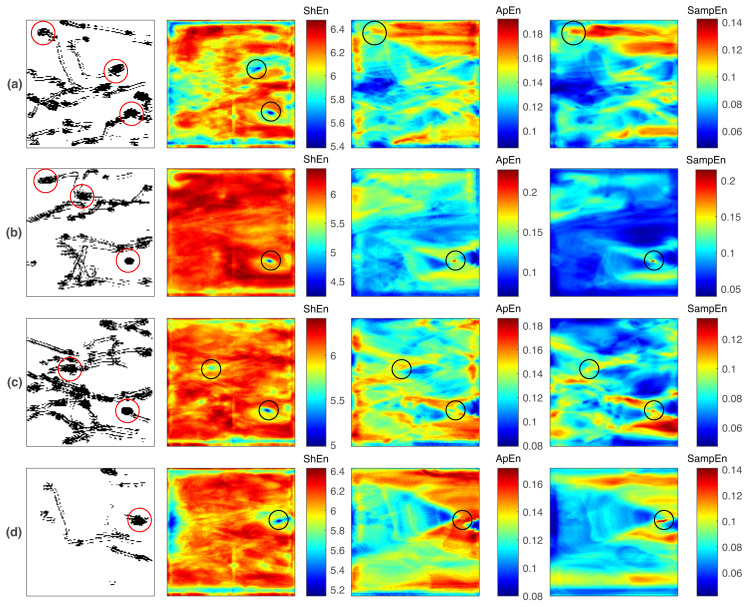
Characterization of multiple-reentry dynamics resulting from the implementation of stimulation protocol (i) and the cAF4 condition. From (**a**) to (**d**), the maps correspond to four consecutive 1000 ms intervals. The PS trajectory is shown in the first column; ShEn, ApEn, and SampEn maps are shown from the second to the last column, respectively. Red circles in the PS maps mark the occurrence of reentries. Black circles in the entropy maps mark detections matching the reentries defined in the PS maps.

**Figure 8:**
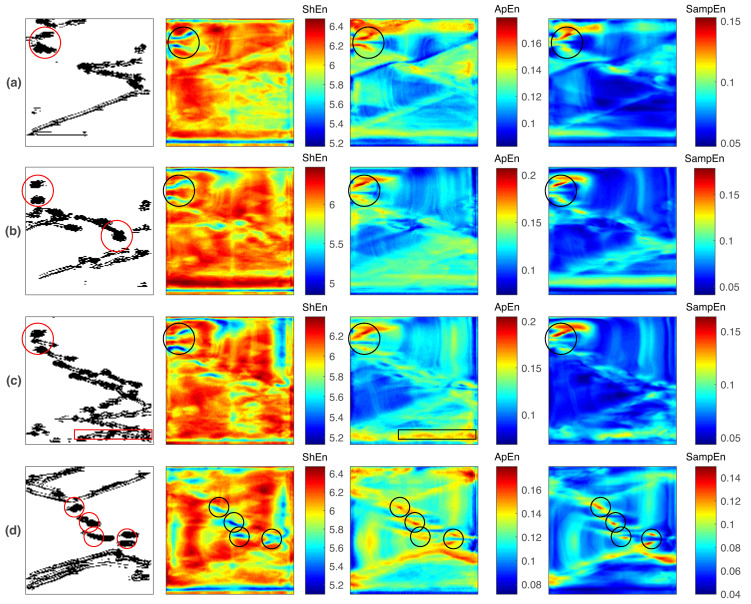
Characterization of reentry breakup dynamics resulting from the implementation of stimulation protocol (i) and the cAF5 condition. From (**a**) to (**d**), the maps correspond to four consecutive 1000 ms intervals. The PS trajectory is shown in the first column; ShEn, ApEn, and SampEn maps are shown from the second to the last column, respectively. Red contours in the PS maps mark the occurrence of reentries. Black contours in the entropy maps mark reentries detections.

Unlike the previous studied conditions, cAF4 and cAF5 do not generate persistent reentries. Complex propagation patterns and multiple small reentries coexist during the entire simulation. The corresponding PS maps, shown in the first column in Figure 7 (cAF4) and Figure 8 (cAF5), depict several PS trajectories related to small transient reentries and other fibrillatory patterns. In order to discriminate the reentries within the PS maps, they are visually identified in the action potential propagation map and then marked with red circles in the PS map. For this purpose, a reentry is defined as a propagation wave that rotates for more than three spins during a 1000 ms window of observation. The entropy analysis was implemented by applying a moving window of 1000 samples without overlapping, obtaining four entropy maps describing the dynamics of the whole episode. These results are shown in Figure 7 (cAF4) and Figure 8 (cAF5), where each row, from top to bottom, depicts 1000 ms consecutive intervals. In the entropy maps, detections matching the reentries defined in the PS maps are marked with black circles.

The PS maps reveal that, under the cAF4 condition (first column of Figure 7), nine reentries are detected. The entropy maps have different representations of the reentry dynamics: while the ShEn maps highlight the rotors by low ShEn values, the ApEn and SampEn maps highlight the rotors by high entropy values. Despite observing high ApEn and SampEn values and low ShEn values in some areas of the entropy maps that agree with the reentries marked in the PS maps, there are other regions with those characteristics that represent false detections. The poor performance of the entropy maps could be related to the ephemeral lifespan of reentries, since their duration is shorter than the 1000 ms observation window.

The PS maps for cAF5 conditions (the first column of Figure 8) reveal several PS trajectories, but there are fewer compared with those for cAF4. During 4 s of simulation, nine reentries are observed. A figure-of-eight reentry is located at the left superior corner of the domain, and it endures for three consecutive observation windows (Figure 8a–c). This mechanism is detected by the all of the entropy maps by low ShEn values and high ApEn and SampEn values. During the last observation window, Figure 8d, three rotors and a figure-of-eight reentry are observed, and the entropy maps are able to detect them. Particular behaviors are observed: in the second observation window, Figure 8b, one rotor is missed by all entropy maps. In the third observation window, Figure 8c, there is a drifting rotor that is detected only by the ApEn map. In this fibrillation condition, the entropy maps show better reentry detection performance compared with the results depicted in Figure 7. The high ApEn and SampEn areas and low ShEn areas agree with the majority of reentries marked in the PS maps. Such a positive performance could be related to the fact that the reentries are sustained for a time close to or greater than the 1000 ms observation window.

## 5. Discussion

In this work, reentry dynamics in simulated AF episodes are studied using ShEn, ApEn, and SampEn maps. These entropy metrics are implemented to quantify the unipolar EGM irregularity generated by reentry propagation patterns in three distinct fibrillation scenarios. In this manner, a relation between EGM fractionation phenomena and arrhythmogenic mechanisms, such as functional reentries, can be established. The principal findings are summarized as follows:

- ApEn and SampEn present similar behaviors in the characterization of reentry dynamics, with ShEn differing from these results. The ApEn and SampEn maps consistently highlight the reentry core region by high entropy values in all of the studied cases, while the ShEn maps mark the reentries by high or low entropy values, depending on the fibrillation dynamics.
- When no other fibrillation mechanism is present within the designed domain, the ApEn and SampEn maps better match the reentry core region by high entropy values, while ShEn is less specific since it highlights a broader area.
- When multiple reentries coexist with other fibrillatory patterns, reentry identification can be challenging for all three cases. Under such conditions, it is hypothesized that the reentries need to endure for a time that is comparable to the observation window so that the entropy maps can detect them.

The rotor hypothesis postulates that atrial fibrillatory activity is sustained by rapid and successive wavefronts that emanate from one or several relatively stable rotors [40]. Hence, rotor ablation could lead to AF termination. Therefore, the identification and characterization of functional reentries can be a valuable tool for AF treatment. In this work, such task was achieved by applying entropy measures to quantify EGM irregularity and generate entropy electroanatomical maps. The results suggest that the entropy maps can detect a functional reentry if the mechanism is sustained over a period of time that is close to, or longer than the duration of the observation window. Otherwise, false detections arise. Therefore, entropy maps seem to fulfill the rotor hypothesis criteria: a functional reentry requires time stability in order to sustain an AF. A previous in-silico study assessed ApEn maps as an ablation guiding tool [38]. The study found that the application of an ablation line that crosses the high-ApEn zone (corresponding to a rotor tip trajectory) terminates the AF episode. Although that study considered only a quasi-stable rotor (as in the episode shown in Figure 2), the results encourage the pursuit of investigations for determining whether these high-entropy sites can yield successful ablation in distinct AF propagation scenarios.

The solutions for rotor mapping during AF have reached the clinical context. The focal impulse and rotor modulation (FIRM) approach is a mapping procedure for guiding AF ablation [50]. The procedure has been applied to patients with reportedly high success rates of rotor ablation resulting in the termination of an AF [51]. Although it is known that this approach is based on phase maps, there is no detailed information about the signal processing methods implemented in the FIRM algorithm [69]. Nevertheless, a recent study conducted by Benharash et al. reports that the EGMs targeted as a rotor core by the FIRM approach are not quantitatively different from the EGMs in other atrial zones after a ShEn analysis [70]. From the results obtained in the present in-silico work, there are two scenarios that could be related to the mentioned FIRM outcome: first, the EGMs corresponding to the core of the reentries have a distinguishing characteristic that can be quantified using ApEn and SampEn but not with ShEn (Figure 3 and Figure 5). This occurs when a single reentry (either a rotor or a figure-of-eight reentry) is within the designed atrial domain. This scenario agrees with the findings of Benharash et al. [70] and could imply that the FIRM detections are related to rotors and that the ShEn measure is not suitable for describing rotor dynamics. Second, when multiple reentries and fibrillatory propagations are found simultaneously during an AF simulation, the three assessed entropy measures are able to discriminate the reentries (Figure 7), but they also miss the detection of other reentries and present false detections (Figure 8). Such poor performance from the use of entropy measures agrees with Benharash et al.’s observation of rotor cores having EGM signals that do not differ quantitatively from their surroundings. This would imply that the FIRM detections are not related to arrhythmogenic mechanisms. Furthermore, the ephemeral reentries shown in Figure 7 could not be substrates sustaining the AF due their short lifespan, while the reentries observed in the Figure 8 may be more relevant to the AF dynamics because they present some degree of temporal stability. The understanding of such dynamics could be pursued by implementing the recently proposed causality analysis of AF that enables the determination of the dominant conduction regions in AF maintenance [46,47,48]. A detailed study is needed that aims to identify functional reentries, to determine which reentries are relevant to sustaining the AF, and to describe their quantitative behavior.

Phase map analysis is a tool that is broadly used to characterize fibrillatory dynamics in controlled conditions, such computational simulations. However, its implementation in clinical procedures would require high spatial resolution [71] and noiseless EGM signals with clear activation waveforms [72]. Such conditions are difficult to accomplish due to the limitations of the current electrophysiological technology. Additionally, EGMs present a complex morphology in fibrillatory conditions. Even though the EGM can be preprocessed to reduce noise and artifacts, the computation time would extend the clinical procedure, hindering the reproducibility of the results [70,73]. Therefore, the motivation to develop new tools for EGM and fibrillation analysis arises. In the case of the entropy analysis, these measures are more robust to noisy signals and can be applied to quantitatively assess the complexity of the EGM instead of looking for smooth and clear atrial activations, which are hard to record in fibrillatory conditions due to the disorganized propagation underlying the arrhythmia.

Under the simulation conditions implemented in this work, the ShEn maps mark the reentries through high or low entropy values, depending on the fibrillation dynamics. Specifically, for the single-rotor case, high ShEn values are observed in the reentry core regions and at the periphery, resulting in a broad high-ShEn area (the first column in Figure 3). The same behavior occurs for the figure-of-eight reentries (the first column in Figure 5), where broad high-ShEn areas containing the reentry cores are observed. Hence, the ShEn maps perform poorly for accurately detecting the reentry cores by means of high entropy values. Signal irregularity using ShEn is estimated on the basis of the shape of the distribution of its amplitude values. Although EGM morphologies at the reentry core differ from those at the periphery, the obtained results suggest that the corresponding EGM amplitude distributions remain similar, preventing the discrimination between the reentry core and its periphery. For the fibrillatory episodes with multiple and other complex propagation patterns (the first column in Figure 7 and Figure 8), low ShEn values are found at the reentry cores sites. This reversed behavior indicates that the propagation patterns are distinct from functional reentries but coexist with them, and they generate EGMs whose amplitude distribution is broader than the EGMs from the reentry cores. There are studies reporting that the reentry core is related to high ShEn values [36,39,52,74] and also to low ShEn values [75,76]. Moreover, the outlined dual ShEn behavior during reentry detection has been recently described in a study in which high-ShEn areas determined the reentry positions when two rotors coexisted, while low- ShEn areas determined the reentry positions when three rotors coexisted [33]. An open question thus remains as to whether this feature of the ShEn measure has a clinical meaning or indicates the insufficient ability to describe AF dynamics. In this regard, the ApEn and SampEn maps present consistent results in the three studied scenarios: the reentry cores are described by high entropy values. They measure morphological irregularity using the previous knowledge of the amplitude values. These properties allow ApEn and SampEn to better quantify the differences between EGM at the reentry core and EGM at the periphery by high entropy values. This is an attractive feature for possible translation into clinical practice. Further studies are needed to determine and fully understand the relation between these entropy measures and fibrillation dynamics.

## Abbreviations

The following abbreviations are used in this manuscript:

AF	atrial fibrillation
ApEn	approximate entropy
cAF	chronic atrial fibrillation
CFAE	complex fractionated atrial electrograms
EGM	electrogram(s)
pAF	paroxysmal atrial fibrillation
PS	phase singularity
SampEn	sample entropy
SD	standard deviation
ShEn	Shannon entropy

## References

[1] Kirchhof P., Benussi S., Kotecha D., Ahlsson A., Atar D., Casadei B., Castella M., Diener H.C., Heidbuchel H., Hendriks J. (2016). 2016 ESC Guidelines for the management of atrial fibrillation developed in collaboration with EACTS. Europace.

[2] Björck S., Palaszewski B., Friberg L., Bergfeldt L. (2013). Atrial fibrillation, stroke risk, and warfarin therapy revisited: A population-based study. Stroke.

[3] Zaman J.A.B., Harling L., Ashrafian H., Darzi A., Gooderham N., Athanasiou T., Peters N.S. (2016). Post-operative atrial fibrillation is associated with a pre-existing structural and electrical substrate in human right atrial myocardium. Int. J. Cardiol..

[4] Cantú C., True Hills M., Massaro A., Goto S., Hu H.H., Quek D.K., Sim K.H., Tse H.F., Zhang S., Benbow A. Atrial Fibrillation-Related Stroke across Latin America: A Preventable Problem. https://www.stopafib.org/downloads/News436-LatAm-Prevent.pdf.

[5] Corradi D. (2014). Atrial fibrillation from the pathologist’s perspective. Cardiovasc. Pathol. Off. J. Soc. Cardiovasc. Pathol..

[6] Haïssaguerre M., Jaïs P., Shah D.C., Takahashi A., Hocini M., Quiniou G., Garrigue S., Le Mouroux A., Le Métayer P., Clémenty J. (1998). Spontaneous Initiation of Atrial Fibrillation by Ectopic Beats Originating in the Pulmonary Veins. N. Engl. J. Med..

[7] Kallergis E.M., Goudis C.A., Vardas P.E. (2014). Atrial fibrillation: A progressive atrial myopathy or a distinct disease?. Int. J. Cardiol..

[8] Martins R.P., Kaur K., Hwang E., Ramirez R.J., Willis B.C., Filgueiras-Rama D., Ennis S.R., Takemoto Y., Ponce-Balbuena D., Zarzoso M. (2014). Dominant frequency increase rate predicts transition from paroxysmal to long-term persistent atrial fibrillation. Circulation.

[9] Li X., Salinet J.L., Almeida T.P., Vanheusden F.J., Chu G.S., Ng G.A., Schlindwein F.S. (2017). An interactive platform to guide catheter ablation in human persistent atrial fibrillation using dominant frequency, organization and phase mapping. Comput. Methods Progr. Biomed..

[10] Sasaki N., Watanabe I., Okumura Y., Nagashima K., Kogawa R., Sonoda K., Iso K., Takahashi K., Arai M., Watanabe R. (2017). Complex fractionated atrial electrograms, high dominant frequency regions, and left atrial voltages during sinus rhythm and atrial fibrillation. J. Arrhythmia.

[11] Ng J., Goldberger J.J. (2007). Understanding and interpreting dominant frequency analysis of AF electrograms. J. Cardiovasc. Electrophysiol..

[12] Stiles M.K., Brooks A.G., Kuklik P., John B., Dimitri H., Lau D.H., Wilson L., Dhar S., Roberts-Thomson R.L., Mackenzie L. (2008). The relationship between electrogram cycle length and dominant frequency in patients with persistent atrial fibrillation. J. Cardiovasc. Electrophysiol..

[13] Nademanee K., McKenzie J., Kosar E., Schwab M., Sunsaneewitayakul B., Vasavakul T., Khunnawat C., Ngarmukos T. (2004). A new approach for catheter ablation of atrial fibrillation: Mapping of the electrophysiologic substrate. J. Am. Coll. Cardiol..

[14] Ammar-Busch S., Reents T., Knecht S., Rostock T., Arentz T., Duytschaever M., Neumann T., Cauchemez B., Albenque J.P., Hessling G. (2018). Correlation between atrial fibrillation driver locations and complex fractionated atrial electrograms in patients with persistent atrial fibrillation. PACE-Pacing Clin. Electrophysiol..

[15] Martin C.A., Curtain J.P., Gajendragadkar P.R., Begley D.A., Fynn S.P., Grace A.A., Heck P.M., Virdee M.S., Agarwal S. (2018). Ablation of Complex Fractionated Electrograms Improves Outcome in Persistent Atrial Fibrillation of Over 2 Year’s Duration. J. Atr. Fibrillation.

[16] Kochhäuser S., Verma A., Dalvi R., Suszko A., Alipour P., Sanders P., Champagne J., Macle L., Nair G.M., Calkins H. (2017). Spatial Relationships of Complex Fractionated Atrial Electrograms and Continuous Electrical Activity to Focal Electrical Sources: Implications for Substrate Ablation in Human Atrial Fibrillation. JACC Clin. Electrophysiol..

[17] Ammar-Busch S., Bourier F., Reents T., Semmler V., Telishevska M., Kathan S., Hofmann M., Hessling G., Deisenhofer I. (2017). Ablation of Complex Fractionated Electrograms With or Without ADditional LINEar Lesions for Persistent Atrial Fibrillation (The ADLINE Trial). J. Cardiovasc. Electrophysiol..

[18] Seitz J., Bars C., Ferracci A., Maluski A., Penaranda G., Theodore G., Faure J., Bremondy M., Curel L., Beurtheret S. (2016). Electrogram Fractionation-Guided Ablation in the Left Atrium Decreases the Frequency of Activation in the Pulmonary Veins and Leads to Atrial Fibrillation Termination: Pulmonary Vein Modulation Rather Than Isolation. JACC Clin. Electrophysiol..

[19] Oketani N., Seitz J., Salazar M., Pisapia A., Kalifa J., Smit J.J., Nademanee K. (2016). Ablation of complex fractionated electrograms is useful for catheter ablation of persistent atrial fibrillation: Protagonist point of view. Heart Rhythm.

[20] Verma A., Jiang C.Y., Betts T., Ghen J., Deisenhofer I., Mantovan R., Macle L., Morillo C.A., Haverkamp W., Weerasooriya R. (2015). Approaches to Catheter Ablation for Persistent Atrial Fibrillation Atul. Int. J. Mech. Mechatron. Eng..

[21] Chen J., Lin Y., Chen L., Yu J., Du Z., Li S., Yang Z., Zeng C., Lai X., Lu Q. (2014). A decade of complex fractionated electrograms catheter-based ablation for atrial fibrillation: Literature analysis, meta-analysis and systematic review. IJC Heart Vessels.

[22] Dixit S., Marchlinski F.E., Lin D., Callans D.J., Bala R., Riley M.P., Garcia F.C., Hutchinson M.D., Ratcliffe S.J., Cooper J.M. (2012). Randomized ablation strategies for the treatment of persistent atrial fibrillation RASTA study. Circ. Arrhythmia Electrophysiol..

[23] Berenfeld O., Jalife J. (2011). Complex Fractionated Atrail Electrograms Is this the Beast to Tame in AF. Circ. Arrhythmia Electrophysiol..

[24] Adragão P., Carmo P., Cavaco D., Carmo J., Ferreira A., Moscoso Costa F., Carvalho M.S., Mesquita J., Quaresma R., Belo Morgado F. (2017). Relationship between rotors and complex fractionated electrograms in atrial fibrillation using a novel computational analysis. Revista Portuguesa de Cardiologia.

[25] Almeida T.P., Schlindwein F.S., Salinet J., Li X., Chu G.S., Tuan J.H., Stafford P.J., André Ng G., Soriano D.C. (2018). Characterization of human persistent atrial fibrillation electrograms using recurrence quantification analysis. Chaos.

[26] Cirugeda-Roldán E., Molina Picó A., Novák D., Cuesta-Frau D., Kremen V. (2018). Sample Entropy Analysis of Noisy Atrial Electrograms during Atrial Fibrillation. Comput. Math. Methods Med..

[27] Navoret N., Jacquir S., Laurent G., Binczak S. (2013). Detection of complex fractionated atrial electrograms using recurrence quantification analysis. IEEE Trans. Bio-Med. Eng..

[28] Bonizzi P., Zeemering S., Karel J.M., Di Marco L.Y., Uldry L., Van Zaen J., Vesin J.M., Schotten U. (2015). Systematic comparison ofnon-invasive measures for the assessment ofatrial fibrillation complexity: A step forward towards standardization ofatrial fibrillation electrogram analysis. Europace.

[29] Orozco-Duque A., Novak D., Kremen V., Bustamante J. (2015). Multifractal analysis for grading complex fractionated electrograms in atrial fibrillation. Physiol. Meas..

[30] Cirugeda-Roldán E., Novak D., Kremen V., Cuesta-Frau D., Keller M., Luik A., Srutova M. (2015). Characterization of complex fractionated atrial electrograms by sample entropy: An international multi-center study. Entropy.

[31] Ugarte J., Orozco-Duque A., Tobón C., Kremen V., Novak D., Saiz J., Oesterlein T., Schmitt C., Luik A., Bustamante J. (2014). Dynamic approximate entropy electroanatomic maps detect rotors in a simulated atrial fibrillation model. PLoS ONE.

[32] Orozco-Duque A., Tobón C., Ugarte J., Morillo C., Bustamante J. (2018). Electroanatomical mapping based on discrimination of electrograms clusters for localization of critical sites in atrial fibrillation. Prog. Biophys. Mol. Biol..

[33] Aronis K.N., Ashikaga H. (2018). Impact of number of co-existing rotors and inter-electrode distance on accuracy of rotor localization. J. Electrocardiol..

[34] Song J.S., Wi J., Lee H.J., Hwang M., Lim B., Kim T.H., Uhm J.S., Joung B., Lee M.H., Seo J.W. (2017). Role of atrial wall thickness in wave-dynamics of atrial fibrillation. PLoS ONE.

[35] Duque S., Orozco-Duque A., Kremen V., Novak D., Tobón C., Bustamante J. (2017). Feature subset selection and classification of intracardiac electrograms during atrial fibrillation. Biomed. Signal Process. Control.

[36] Hwang M., Song J.S., Lee Y.S., Li C., Shim E.B., Pak H.N. (2016). Electrophysiological rotor ablation in in-silico modeling of atrial fibrillation: Comparisons with dominant frequency, shannon entropy, and phase singularity. PLoS ONE.

[37] Orozco-Duque A., Bustamante J., Castellanos-Dominguez G. (2016). Semi-supervised clustering of fractionated electrograms for electroanatomical atrial mapping. BioMed. Eng. Online.

[38] Ugarte J.P., Tobón C., Orozco-Duque A., Becerra M.A., Bustamante J. (2015). Effect of the electrograms density in detecting and ablating the tip of the rotor during chronic atrial fibrillation: An in silico study. Europace.

[39] Ganesan A.N., Kuklik P., Gharaviri A., Brooks A., Chapman D., Lau D.H., Roberts-Thomson K.C., Sers P. (2014). Origin and characteristics of high Shannon entropy at the pivot of locally stable rotors: Insights from computational simulation. PLoS ONE.

[40] Jalife J. (2003). Rotors and spiral waves in atrial fibrillation. J. Cardiovasc. Electrophysiol..

[41] Allessie M., de Groot N. (2014). CrossTalk opposing view: Rotors have not been demonstrated to be the drivers of atrial fibrillation. J. Physiol..

[42] Hansen B.J., Zhao J., Csepe T.A., Moore B.T., Li N., Jayne L.A., Kalyanasundaram A., Lim P., Bratasz A., Powell K.A. (2015). Atrial fibrillation driven by micro-anatomic intramural re-entry revealed by simultaneous sub-epicardial and sub-endocardial optical mapping in explanted human hearts. Eur. Heart J..

[43] Zhao J., Hansen B.J., Csepe T.A., Lim P., Wang Y., Williams M., Mohler P.J., Janssen P.M., Weiss R., Hummel J.D. (2015). Integration of High-Resolution Optical Mapping and 3-Dimensional Micro-Computed Tomographic Imaging to Resolve the Structural Basis of Atrial Conduction in the Human Heart. Circ. Arrhythmia Electrophysiol..

[44] De Groot N., Van Der Does L., Yaksh A., Lanters E., Teuwen C., Knops P., Van De Woestijne P., Bekkers J., Kik C., Bogers A. (2016). Direct Proof of Endo-Epicardial Asynchrony of the Atrial Wall During Atrial Fibrillation in Humans. Circ. Arrhythmia Electrophysiol..

[45] Zhao J., Hansen B.J., Wang Y., Csepe T.A., Sul L.V., Tang A., Yuan Y., Li N., Bratasz A., Powell K.A. (2017). Three-dimensional integrated functional, structural, and computational mapping to define the structural “fingerprints” of heart-specific atrial fibrillation drivers in human heart ex vivo. J. Am. Heart Assoc..

[46] Biton Y., Rabinovitch A., Braunstein D., Aviram I., Campbell K., Mironov S., Herron T., Jalife J., Berenfeld O. (2018). Causality analysis of leading singular value decomposition modes identifies rotor as the dominant driving normal mode in fibrillation. Chaos.

[47] Cervigón R., Castells F., Gómez-Pulido J.M., Pérez-Villacastín J., Moreno J. (2018). Granger causality and Jensen-Shannon divergence to determine dominant atrial area in Atrial fibrillation. Entropy.

[48] Rodrigo M., Climent A.M., Liberos A., Calvo D., Fernández-Avilés F., Berenfeld O., Atienza F., Guillem M.S. (2016). Identification of Dominant Excitation Patterns and Sources of Atrial Fibrillation by Causality Analysis. Ann. Biomed. Eng..

[49] Miller J.M., Kalra V., Das M.K., Jain R., Garlie J.B., Brewster J.A., Dandamudi G. (2017). Clinical Benefit of Ablating Localized Sources for Human Atrial Fibrillation: The Indiana University FIRM Registry. J. Am. Coll. Cardiol..

[50] Narayan S.M., Patel J., Mulpuru S., Krummen D.E. (2012). Focal impulse and rotor modulation ablation of sustaining rotors abruptly terminates persistent atrial fibrillation to sinus rhythm sith elimination on follow-up A video case study. Heart Rhythm.

[51] Narayan S.M., Shivkumar K., Krummen D.E., Miller J.M., Rappel W.J. (2013). Panoramic electrophysiological mapping but not electrogram morphology identifies stable sources for human atrial fibrillation: Stable atrial fibrillation rotors and focal sources relate poorly to fractionated electrograms. Circ. Arrhythmia Electrophysiol..

[52] Ganesan A.N., Kuklik P., Lau D.H., Brooks A.G., Baumert M., Lim W.W., Thanigaimani S., Nayyar S., Mahajan R., Jonathan M. (2013). Bipolar Electrogram Shannon Entropy at Sites of Rotational Activation: Implications for Abaltion of Atrial Fibrillation. Circ. Arrhythmia Electrophysiol..

[53] Courtemanche M., Ramirez R.J., Nattel S. (1998). Ionic mechanisms underlying human atrial action potential properties: Insights from a mathematical model. Am. J. Physiol..

[54] Fenton F.H., Cherry E.M., Hastings H.M., Evans S.J. (2002). Multiple mechanisms of spiral wave breakup in a model of cardiac electrical activity. Chaos.

[55] Kneller J., Zou R., Vigmond E.J., Wang Z., Leon L.J., Nattel S. (2002). Cholinergic Atrial Fibrillation in a Computer Model of a Two-Dimensional Sheet of Canine Atrial Cells With Realistic Ionic Properties. Circ. Res..

[56] Niwano S., Wakisaka Y., Kojima J., Yumoto Y., Inuo K., Hara H., Saito J., Niwano H., Izumi T. (2003). Monitoring the Progression of the Atrial Electrical Remodeling in Patients With Paroxysmal Atrial Fibrillation. Circ. J..

[57] Nattel S., Burstein B., Dobrev D. (2008). Atrial Remodeling and Atrial Fibrillation. Circ. Arrhythmia Electrophysiol..

[58] Van Wagoner D.R., Pond A.L., McCarthy P.M., Trimmer J.S., Nerbonne J.M. (1997). Outward K+ Current Densities and Kv1.5 Expression Are Reduced in Chronic Human Atrial Fibrillation. Circ. Res..

[59] Bosch R.F., Zeng X., Grammer J.B., Popovic K., Mewis C., Kühlkamp V. (1999). Ionic mechanisms of electrical remodeling in human atrial fibrillation. Cardiovasc. Res..

[60] Dobrev D., Graf E., Wettwer E., Himmel H., Hála O., Doerfel C., Christ T., Schüler S., Ravens U. (2001). Molecular Basis of Downregulation of G-Protein–Coupled Inward Rectifying K+ Current (IK,ACh) in Chronic Human Atrial Fibrillation. Circulation.

[61] Workman A.J., Kane K.A., Rankin A.C. (2001). The contribution of ionic currents to changes in refractoriness of human atrial myocytes associated with chronic atrial fibrillation. Cardiovasc. Res..

[62] Ugarte J.P., Tobón C., Lopes A.M., Tenreiro Machado J.A. (2018). Atrial rotor dynamics under complex fractional order diffusion. Front. Physiol..

[63] Hansson A., Holm M., Blomström P., Johansson R., Lührs C., Brandt J., Olsson S.B. (1998). Right atrial free wall conduction velocity and degree of anisotropy in patients with stable sinus rhythm studied during open heart surgery. Eur. Heart J..

[64] Bueno-Orovio A., Kay D., Burrage K. (2014). Fourier spectral methods for fractional-in-space reaction-diffusion equations. BIT Numer. Math..

[65] Pincus S.M. (1991). Approximate entropy as a measure of system complexity. Proc. Natl. Acad. Sci. USA.

[66] Richman J.S., Moorman J.R. (2000). Physiological time-series analysis using approximate entropy and sample entropy Physiological time-series analysis using approximate entropy and sample entropy. Am. J. Physiol. Heart Circ. Physiol..

[67] Kuklik P., Zeemering S., Maesen B., Maessen J., Crijns H.J., Verheule S., Ganesan A.N., Schotten U. (2015). Reconstruction of instantaneous phase of unipolar atrial contact electrogram using a concept of sinusoidal recomposition and hilbert transform. IEEE Trans. Biomed. Eng..

[68] Bray M.A., Lin S.F., Aliev R.R., Roth B.J., Wikswo J.P. (2001). Experimental and theoretical analysis of phase singularity dynamics in cardiac tissue. J. Cardiovasc. Electrophysiol..

[69] Baumert M., Sanders P., Ganesan A. (2016). Quantitative-Electrogram-Based Methods for Guiding Catheter Ablation in Atrial Fibrillation. Proc. IEEE.

[70] Benharash P., Buch E., Frank P., Share M., Tung R., Shivkumar K., Mandapati R. (2015). Quantitative Analysis of Localized Sources Identified by Focal Impulse and Rotor Modulation Mapping in Atrial Fibrillation. Circ. Arrhythmia Electrophysiol..

[71] Roney C.H., Cantwell C.D., Bayer J.D., Qureshi N.A., Lim P.B., Tweedy J.H., Kanagaratnam P., Peters N.S., Vigmond E.J., Ng F.S. (2017). Spatial resolution requirements for accurate identification of drivers of atrial fibrillation. Circ. Arrhythmia Electrophysiol..

[72] Clayton R.H., Nash M.P. (2015). Analysis of cardiac fibrillation using phase mapping. Card. Electrophysiol. Clin..

[73] Buch E., Share M., Tung R., Benharash P., Sharma P., Koneru J., Mandapati R., Ellenbogen K.A., Shivkumar K. (2016). Long-term clinical outcomes of focal impulse and rotor modulation for treatment of atrial fibrillation: A multicenter experience. Heart Rhythm.

[74] Arunachalam S.P., Mulpuru S.K., Friedman P.A., Tolkacheva E.G. (2015). Feasibility of visualizing higher regions of Shannon entropy in atrial fibrillation patients. Conf. Proc. IEEE Eng. Med. Biol. Soc..

[75] Annoni E.M., Arunachalam S.P., Kapa S., Mulpuru S.K., Friedman P.A., Tolkacheva E.G. (2018). Novel quantitative analytical approaches for rotor identification and associated implications for mapping. IEEE Trans. Biomed. Eng..

[76] Arunachalam S., Kapa S., Mulpuru S., Friedman P., Tolkacheva E. Rotor pivot point identification using recurrence period density entropy. Proceedings of the 54th Annual Rocky Mountain Bioengineering Symposium.

